# Neuroprotective Effect of Low Mean Arterial Pressure on Postoperative Cognitive Deficit Attenuated by Prolonged Coronary Artery Bypass Time: A Meta-Analysis

**DOI:** 10.21470/1678-9741-2018-0263

**Published:** 2019

**Authors:** Farshad Hasanzadeh Kiabi, Aria Soleimani, Mohammad Reza Habibi

**Affiliations:** 1 Department of Anesthesiology, Faculty of Medicine, Mazandaran University of Medical Sciences, Sari, Iran.

**Keywords:** Arterial Pressure, Cardiopulmonary Bypass, Coronary Artery Disorders, Neuroprotective Agents, Cognitive Disorders, Meta-Analysis

## Abstract

**Introduction:**

The true influence of the low mean arterial pressure (low MAP) during coronary artery bypass grafting (CABG) on the development of postoperative cognitive deficit (POCD) remains controversial. We aimed to perform a meta-analysis and meta-regression to determine the effect of low MAP on POCD, as well as moderator variables between low MAP and POCD.

**Methods:**

The Web of Science, PubMed database, Scopus and the Cochrane Library database (up to June 2018) were searched and retrieved articles systematically reviewed. Only randomized controlled trials (RCTs) comparing maintenance of low MAP (<80 mmHg) and high MAP (>80 mmHg) during cardiopulmonary bypass (CPB) were included in our final review. Statistical analysis of the risk ratio (RR) and corresponding 95% confidence interval (CI) was used to report the overall effect. The overall effect and meta-regression analysis were done using Mantel-Haenszel risk ratio (MHRR) and the corresponding 95% confidence interval (CI).

**Results:**

A total of 731 patients in three RCTs were included in this study. POCD occurred in 6.4% of all cases. Maintenance of low MAP did not reduce the occurrence of POCD (MHRR 1.012 [95% CI 0.277-3.688]; *Z*=0.018; *P*=0.986; I^2^=66%). Shorter CPB time reduced the occurrence of POCD regardless of group assignment (MH log risk ratio -0.519 [95% CI -0.949 - -0.089]; *Z*= -2.367; *P*=0.017).

**Conclusion:**

POCD is a common event among CABG patients. The neuroprotective effect of low MAP on POCD was attenuated by the prolonged CPB time.

**Table:** 

Abbreviations, acronyms & symbols				
AUC	= Area under the curve		MHRR	= Mantel-Haenszel risk ratio
CABG	= Coronary artery bypass grafting		OR	= *Odds ratio*
CI	= Confidence interval		POCD	= Postoperative cognitive deficit
CV	= Coefficient of variation		RCTs	= Randomized controlled trials
CPB	= Cardiopulmonary bypass		rLVEF	= Reduced left ventricle ejection fraction
DM	= Diabetes mellitus		RR	= Risk ratio
HTN	= Hypertension		SD	= Standard deviation
MAP	= Mean arterial pressure		WAIS	= Wechsler Adult Intelligence Scale

## INTRODUCTION

Postoperative cognitive deficit (POCD) has long been considered a major complication after cardiac surgery^[1,2]^. Microemboli are assumed to be a key factor in development of brain injury after cardiopulmonary bypass (CPB) surgery^[3]^, but to date the root cause of POCD and relevant risk factors have been poorly differentiated^[4]^. 

Low mean arterial pressure (low MAP) has been one of the most common protocols for decreasing POCD after CPB surgery^[5]^. Low MAP reduces bleeding at the surgical site, the need to cardiotomy suction as well as a reduction in the embolic load^[6]^. Although several reviews have explored the effectiveness of low MAP in reducing POCD among CABG patients, there is certain doubt concerning estimates of MAP threshold to maintain neuroprotection against embolic load^[5-10]^. Pepin et al.^[5]^ concluded that cerebral autoregulation is maintained by MAP as low as 30 mmHg in moderately hypothermic patients. Murphy et al.^[6]^ and Riley^[7]^ suggested that only high-risk patients may require MAP >70 mmHg including patients with advanced atherosclerotic disease of the aorta, hypertension, diabetes and the elderly. Therefore, guidelines are unable to recommend any specific measures for optimal MAP during CPB. In this regard, clinical practice guidelines for MAP management endorsed by American Society of ExtraCorporeal Technology (AmSECT) advised that the intended treatment algorithm for blood pressure management prior to CPB, including acceptable ranges for blood pressure shall be defined by the perfusion care team^[11]^. Although avoidance of high MAP is emphasized in the literature, no threshold MAP during CPB is recommended due to insufficient published evidence^[6]^. Therefore, it is necessary to explore both CPB-related and patient-related factors more precisely to determine moderator variables which play an important role in the association between low MAP and POCD. 

## OBJECTIVE

Due to the potential neuroprotective effect of low MAP, its routine use in anesthesiology, especially CPB surgery and the lack of strong findings from randomized controlled trials (RCTs), we aimed to perform a meta-analysis to examine the effect of low MAP on the POCD and a meta-regression to determine whether moderator variables mediate the relationship between low MAP and POCD.

## METHODS

### Eligibility Criteria

Only RCTs were incorporated into the current meta-analysis: 1) the population comprised CABG patients; 2) there was an intervention group of lower MAP (<80 mmHg) during CPB surgery; 3) there was a control group of higher MAP ≥80 mmHg) during CPB surgery; 4) the main outcome included POCD. We excluded from the meta-analysis studies in which the neuropsychological test battery for patients with CPB surgery to assess cognitive deficit was not administered, and RCTs published in languages other than English. 

### Information Sources

Web of Science, PubMed database, Scopus and the Cochrane Library (until June 2018) were searched. Reference lists of relevant articles were also searched.

### Search

We conducted the search with the following terms: (“coronary artery bypass surgery” OR “cardiopulmonary bypass surgery” OR “thoracic surgery” OR “cardiovascular surgery” OR “cardiac surgery” OR CABG) AND (“cognitive dysfunction” OR “cognitive function” OR “cognitive decline” OR “neurocognitive”) AND (“perfusion pressure” OR “mean arterial pressure” OR “low* MAP” OR “high* MAP” OR “arterial pressure”) AND (“randomised controlled trial” OR “controlled clinical trial” OR “randomized” OR “randomly” OR “trial”). The reference lists of the retrieved studies were also checked, as well as meta-analyses, and systematic reviews on MAP.

### Study Selection

**Fig. 1:**
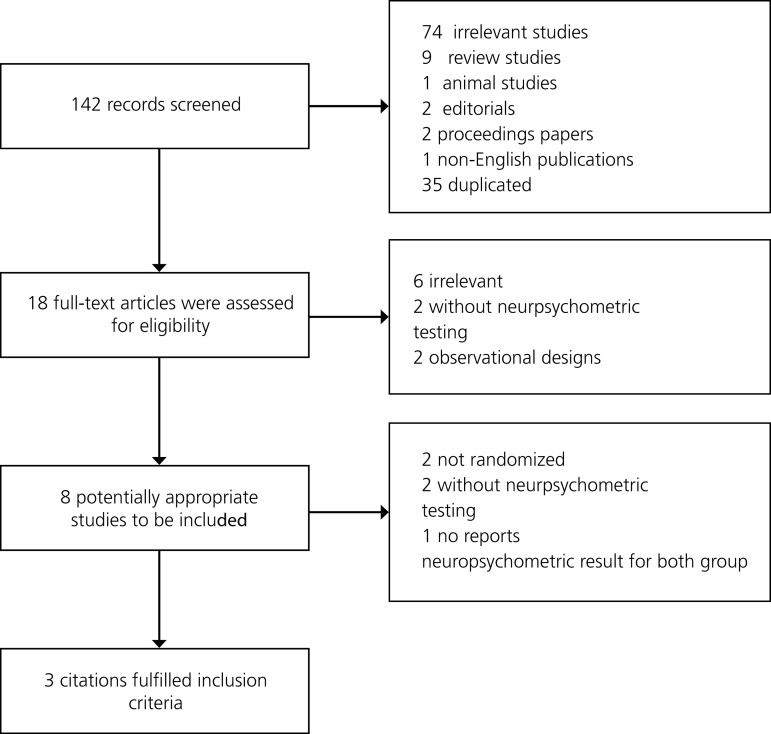
Flow diagram of the selection process for eligible articles.

The study selection process is detailed in Figure 1. First, titles of records were identified and duplicates were removed. Second, abstracts were screened. Third, following a full-text analysis of the selected abstracts, eligible articles were included. 

### Data Items

The primary endpoint was POCD in intervention and control groups. The occurrence of any cognitive deficit after CPB surgery was defined as the primary outcome (a decline >1 SD in the postoperative score in comparison to the preoperative score). The decline must be attributed to one or more neuropsychological tests. The first 30, 30 to 90, and 90 to 360 days are labelled as the very early, early, and late occurrence of cognitive deficit after CPB surgery, respectively^[1]^. The moderator variables including male gender (%), age (%), mean duration of CPB (minutes), mean arterial pressure (MAP), diabetes mellitus (DM) (%), hypertension (HTN) (%), follow-up period (days) and reduced left ventricle ejection fraction (rLVEF) (%) were collected.

### Data Collection Process

Two independent authors extracted the data. Authors solved all disagreements by discussion and consensus. Patient demographic data, surgery characteristics and outcomes were extracted. 

### Risk of Bias in Individual Studies

The Jadad scale, which assesses the quality of published clinical trials based on methods relevant to random assignment, double-blinding, and patient flow, was used to assess the quality of RCTs. This scale scores a maximum of two points for each criterion (randomization and blinding) and one point for the description of withdrawals and dropouts^[12]^. Details of randomization, allocation concealment, blinding of participants and outcomes were detailed in Table 1. Two reviewers verified the included RCTs.

**Table 1:** Baseline characteristics of patients in eligible studies.

	Gold et al.^[16]^, 1995		Charlson et al.^[17]^, 2007		Siepe et al.^[18]^, 2011	
	Intervention	Control	Intervention	Control	Intervention	Control
MAP (mmHg)	50-60	80-100	70-90	80	60-70	80-90
N. of pts	124	124	206	206	48	44
Age, mean (SD)	66.2 (10.1)	65.4 (8.6)	64.3 (13.4)	65.2 (11.1)	65.2 (9.6)	68.7 (8.3)
Male (%)	78 (62.9)	82 (66.12)	146 (71)	146 (71)	40 (83)	34 (77)
Diabetes	23 (18.24)	18 (14.51)	70 (34)	66 (32)	12 (25)	10 (22)
Hypertension	44 (35.48)	56 (45.16)	111 (54)	115 (56)	30 (63)	32 (72)
CPB duration, min, mean (SD)	89.4 (31.5)	84.9 (28.3)	77 (22)	74 (24)	101 (25)	91 (30)
Procedure	CABG with CPB		CABG with CPB		CABG with CPB	
Follow-up (days)	Before surgery, 7 and 180 days postoperatively		The day before surgery and 1-2, 5-6 and 180 days after surgery		The day before and 2 days after surgery	
Dropout (%)	n=11 (4%) at 180 days - Dropouts in low MAP group: 3 refused, 1 lost, 4 moved (5 deaths) - Dropouts in high MAP group: 4 refused, 2 moved (2 deaths)		n=11 (3.1%) at 180 days - 5 (1.5%) refused, 6 (1.6%) lost - 14 (3.4%) deaths - Not reported for each group		n=13 (12.38%) - Dropouts in low MAP group: 4 unable to perform test, 3 additional valves repair - Dropouts in high MAP group: 5 exceeded from targeted perfusion pressure, 1 unable to perform test	
Jadad score	Total score (5): randomization (1); method of randomization (1); blinding (1); method of blinding (1); withdrawal reason (1)		Total score (4): randomization (1); blinding (1); method of blinding (1); withdrawal reason (1)		Total score (3): randomization (1); blinding (1); withdrawal reason (1)	

Intervention: low mean arterial pressure (low MAP); control=high mean arterial pressure (high MAP); CABG=coronary artery bypass surgery; CPB=cardiopulmonary bypass

### Synthesis of Results

*Q* and I^2^ were used to assess statistical heterogeneity among the selected studies. Variables wit*h Q* statistics with *P*-value <0.05 were considered heterogeneous. It means that the amount of total variance is more than we would expect based on within-study error, so the random effect model was assumed. Meta-regression was performed using a random-effect model because there was significant between-study variation^[13]^. The I^2^ statistics represent the degree of heterogeneity and the proportion of variation in treatment that is independent from sampling error. Moderate heterogeneity assumed as I^2^ was 30% to 60%^[14]^.

### Meta-Regression Analysis

The relationship between one or more moderators and a dependent variable was examined by meta-regression analysis^[15]^. The effect size as dependent variable and the moderator variable as independent variable were analyzed to identify potential predictors of effect size. *Q_model_* statistics with *P*-value <0.05 showed that the relationship between moderator variable and treatment effect is stronger than we would expect by chance. Variables with *Z* statistics with *P*-value <0.05 were interpreted as their slope is probably not zero, and the treatment effect is more effective when the moderator variable changes^[13]^. Mantel-Haenszel (MH) log risk ratio was regressed on the following variables: between-group proportion difference of male gender (%), HTN (%) and DM (%), between-group mean difference of age (years), CPB time (minutes) and MAP (mmHg). Difference was defined as the difference between high MAP and low MAP groups (high MAP-low MAP). Negative value of MH log risk ratio is in favour of the neuroprotective effect of low MAP. Data are presented as a mean (standard deviation) for continuous variables and as proportions (%) for categorical variables. 

### Summary Measures

The risk ratio (RR) and its 95% confidence interval (CI) were calculated. Statistical analyses were conducted using the Comprehensive Meta-Analysis v.2 statistical software package (Biostat Inc,; Englewood, New Jersey, USA). Dichotomic results were analyzed using the MH method.

## RESULTS

### Study Selection

From a total of 142 citations identified, 18 articles were reviewed in depth. Three studies (RCTs) with a total of 731 patients were included in the final analyses^[16-18]^. The search strategy is shown in Figure 1. 

### Study Characteristics

All studies had an above moderate score on the Jadad scale; two studies had a high score (Table 1). Among RCTs, the most commonly used scale to examine the severity of cognitive deficit was the Wechsler Adult Intelligence Scale (WAIS). The overall incidence was 6.40% for POCD. The incidence of POCD was 6.37% and 6.44% for low MAP and high MAP groups, respectively. The female patients' frequency was lower than that of male patients in all RCTs. The mean CPB time ranges from 74 to 101 minutes. Also the mean time difference between low MAP and high MAP groups is up to 10 minutes. The proportion of patients with diabetes varied widely (16.5% to 33%), as did the range of patients with hypertension (40.32% to 67.39%). There was no significant difference between the low MAP and the high MAP groups in relation to the baseline characteristics of patients in all RCTs. The primary characteristics of the eligible studies were presented in Table 1. Their data and conclusions are summarized in Table 2. 

**Table 2:** Summary of data and conclusions from eligible studies.

Study	Design	Trial procedure	NP test	Endpoint	Conclusion
Gold et al. ^[16]^, 1995	Parallel groups, randomized, prospective, double-blind trial	- High MAP (80-100) *vs*. low MAP (50-60) - The alpha-stat protocol 17 for blood gas management was used, and body temperature was cooled to 28°C to 30°C. If MAP increased above the target level and was unresponsive to fentanyl or midazolam, sodium nitroprusside infusion was administered. If MAP fell below the target level, phenylephrine was used. If necessary, norepinephrine or metaraminol were added	- Wechsler adult intelligence scale (WAIS-R) - Trail Making A and B - Grooved pegboard test (Pegs) - Boston Naming, Benton Visual Retention and Recognition Test - Controlled Oral Word Association - Mattis-Kovner Verbal Recall and Recognition, and Finger-Tapping Test - Ammons Quick Test - Control tests: CES-D and SF-36 Health Survey	- A decline determined *a priori* by a panel of experts in the postoperative score in comparison to the preoperative score - POCD if occurred in three or more tests	- Higher MAP during CPB can effectively improve outcomes after coronary bypass
Charlson et al.^[17]^, 2007	Parallel groups, prospective, randomized, and double-blinded	- High MAP (80)* vs*. custom MAP (≤90) - In the high MAP group: if MAP was low, a phenylephrine bolus of 5-15 mcg/kg was given to return MAP to the usual usage range; infusions of 10 mg/250 cc phenylephrine were also used, if necessary. If this was inadequate to maintain MAP, norepinephrine and/or metarainol infusions were given. If MAP was above the target MAP, nitroglycerin 1 mcg/kg bolus was given followed by an infusion of 0.5-20 mcg/kg/min as needed. If this was inadequate, sodium nitroprusside 1-15 mcg/kg/min was infused. In the custom group: patients had pressure maintained during full flow in their custom MAP using the same vasoactive drugs employed in the high MAP group	- Wechsler Adult Intelligence Scale (WAIS-R) - Trail Making A and B - Grooved pegboard test (Pegs) - Boston Naming, Benton Visual Retention and Recognition Test - Controlled Oral Word Association - Mattis-Kovner Verbal Recall and Recognition, and Finger-Tapping Test - Ammons Quick Test - Control tests: CES-D and SF-36 Health Survey	- A decline determined *a priori *by a panel of experts in the postoperative score in comparison to the preoperative score - POCD if occurred in three or more tests	- There were no statistically significant differences between the high MAP group and the custom MAP group for cognitive complications
Siepe et al. ^[18]^, 2011	Parallel groups, prospective, randomized, and double-blinded	- High MAP (80-90) *vs*. low MAP (60-70) - Arterial hypertension with norepinephrine (maximal dose 0.4 mg/kg/min) and hypotension with single 5-mg bolus of urapidil (maximal dose 0.1 mg/kg/min)	- Mini-Mental-State Examination (MMSE)	- Any score of 10 points under the preoperative score, together with a positive assessment by a psychologist, was considered delirium tests	Maintenance of perfusion pressure at physiologic levels during normothermic CPB (80-90 mmHg) is associated with less early POCD and delirium

POCD=postoperative cognitive deficit

### Synthesis of Results

**Fig. 2:**
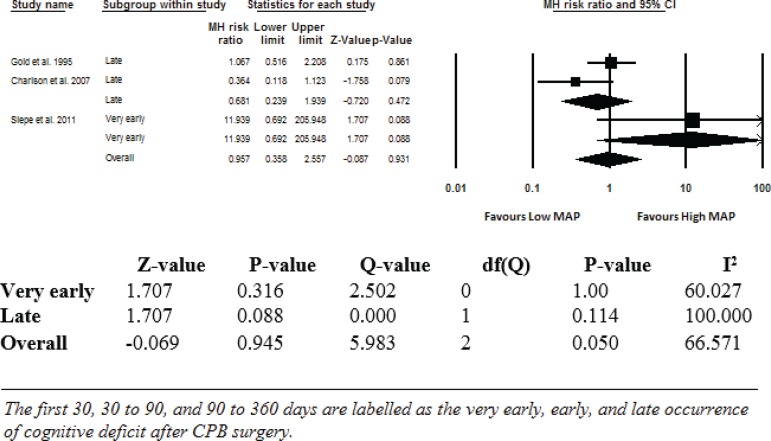
Comparison of patients in low MAP and high MAP.

The overall effect of low MAP was determined by meta-analysis. The MHRR was calculated for the effect of low MAP on POCD as 1.012 (95% CI: 0.277-3.688). The MHRRs for very early and late cognitive deficit were 11.939 (95% CI: 0.692-205.945) and 0.681 (95% CI: 0.239-1.939) (Figure 2). The findings of the pooled analyses, as well as the heterogeneity of the studies, which was found to be 66.57% in total, were summarized in Figure 2. 

### Meta-regression Analysis

**Fig. 3:**
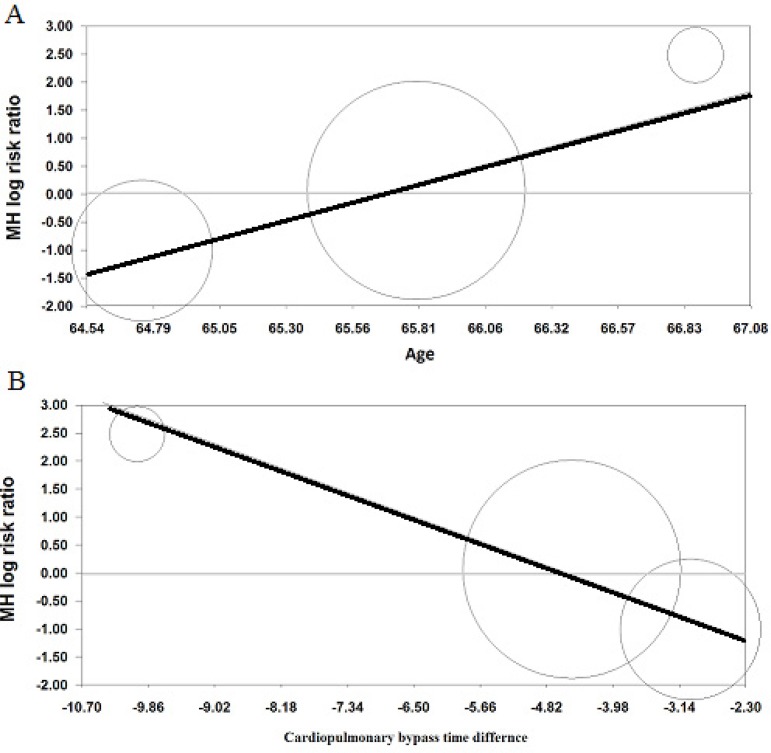
(A) Plot of MH log RR against age. Values in the horizontal line represent mean age in individual studies. Negative values in vertical axis favor the neuroprotective effect of low MAP (below the horizontal gray line). (B) Plot of MH log RR against CPB time difference. Negative values in horizontal axis indicate a longer mean CPB time for the low MAP group (CPB time difference=high MAP – low MAP). Negative values in vertical axis favor the neuroprotective effect of low MAP (below the horizontal gray line).

A meta-regression analysis was performed to determine the overall effect of moderator variables. The MH log RR was calculated for age as 1.281 (95% CI: 0.187-2.375); *Z*-value: 2.296, *P*-value: 0.021, *Q_model_*: 5.273, df: 1, *P*-value: 0.021 (Figure 3A). Figure 3 shows that increasing age is related to a higher occurrence of POCD in the low MAP group. The MH log RR was calculated for between-group CPB time difference as -0.519 (95% CI: -0.949 - -0.089); *Z*-value: -2.367, *P*-value: 0.017, *Q_model_*: 5.603, df: 1, *P*-value: 0.017 (Figure 3B). Figure 3 shows that a lower occurrence of POCD is associated with shorter CPB time in each study, which means that prolonged CPB time in low MAP groups significantly confused the comparison of neuroprotective effect of the studies. Other moderator variables, such as gender, HTN, DM and loss to follow-up, were not significant. 

### Risk of Bias Across Studies

A funnel plot for publication bias analysis is not recommended with less than 10 studies, so it is not shown here^[19]^. Only one of the included studies reported that MAP ranged from 57 to 90 mmHg in the low MAP group^[17]^. In the study by Charlson et al.^[17]^, the MAP target has been set by the pre-bypass MAP in the low MAP group, so patients did not receive MAP higher than baseline MAP during CPB. In high MAP group, MAP kept fixed at 80 mmHg and therefore, no one in high MAP group received MAP <80 mmHg. In contrast, several patients received MAP as low as 57 mmHg in low MAP group. Therefore, it is reasonable to assign patients without hypotension to the high MAP group. 

### Sensitivity Analysis

‘‘Remove-One’’ sensitivity analysis showed that there was no significant association between low MAP and POCD (*P*<0.43) after the exclusion of Charlson et al.^[17]^ study, meaning that overall effect was not biased by the excluded study. In fact, there are no influential studies in any of the analyses based on ‘‘Remove-One’’ sensitivity analysis. In addition, CPB difference for Gold et al. was (-4.5 min) and (-10 min) for Siepe et al.^[18]^ study. CPB difference was (-3 min) for Charlson et al.^[17]^, which is between the values for Siepe et al.^[18]^ and Gold et al.^[16]^, so Charlson et al.^[17]^ could not be a source of bias in meta-regression of CPB time difference on MH log RR (Figure 3B). 

## DISCUSSION

### Summary of Evidence

This is the first meta-regression of the effectiveness of low MAP in reducing POCD after CPB surgery, demonstrating that shorter CPB time had a favorable influence in significantly reducing POCD after CPB surgery. This finding may have critical implications for the therapeutic management of patients undergoing CABG surgery^[1]^.

### The Role of Low MAP

It is routine to keep MAP at a level below the physiological threshold around 50 mmHg^[6,19]^. In most patients, maintaining a sufficient pump flow in the 50 mmHg range does not require any further interventions during CPB^[18]^. The blood supply to the brain is relatively constant because cerebral autoregulation works during CPB when managed either by alpha-stat or pH-stat^[20]^. It is believed that high MAP increases cerebral embolic load as well as the risk of brain injury^[21]^. High MAP results in high CBF, which may increase cerebral embolic load and cerebral edema as well^[22]^. The increasing number of patients with high-risk criteria have raised concerns about the possible indication of high MAP during CPB^[6,7]^. Patients with atherosclerosis of the aorta, hypertension, diabetes, previous stroke and age >70 years are at high risk for POCD^[19]^.

Ono et al.^[20]^ showed that keeping blood pressure below the level of autoregulation is significantly associated with increased morbidity and mortality after cardiac surgery (OR: 1.36; 95% confidence interval: 1.08-1.71; *P*=0.008). The AUC (area under the curve) for low MAP was greater for patients with high morbidity or mortality than for those without (6.5 *vs*. 2.4 mmHg × min/h). They also showed that average MAP during CPB for patients with high morbidity or mortality is not significantly different (75 *vs*. 74 mmHg) from those for patients without high morbidity or mortality. It is worth mentioning, however, that average MAP did not differ between groups, patients with higher morbidity and mortality had significantly longer CPB duration times (133 *vs*. 106 min)^[20]^, implying that CPB time as well as low MAP is strongly associated with morbidity and mortality as well.

In line with other reviews^[5-7]^, meta-analysis showed that low MAP does not reduce POCD after CABG surgery. However, there is no statistically significant difference between the two groups, the incidence of POCD for very early and late was 6.52 and 4.98, respectively, showing that the trend is consistent with that of reported by Brown et al.^[3]^. They found that there is a logarithmic reduction in the frequency of microemboli as well as the embolic load through days spent after CPB (especially in the first few days after CPB surgery). In our study, there is a trend toward lower occurrence of POCD in the low MAP group (6.37% *vs*. 6.44%), suggesting that a confounding variable may interfere with a significant reduction in the occurrence of POCD in the low MAP group. In search of confounding factors that can influence the occurrence of POCD, we further performed meta-regression of moderator variables. Meta-regression analysis showed that there was a significant association between moderators (CBP time difference and age) and POCD.

### The Role of CPB Surgery Time

Meta-regression analysis demonstrated that shorter CPB time is related to a lower rate of POCD in either group, suggesting that CPB time is an independent predictor of POCD. In the regression model, patients with longer surgery time had a significantly increased risk for the occurrence of POCD regardless of the type of group. Bearing in mind that no study had a shorter surgery time for the low MAP group, it may explain why the studies' findings have been inconclusive across all previous reviews. Figure 3 shows that the protective effect of low MAP can only resist up to 4 minutes of longer mean duration of high MAP. Other studies also support this finding, implying that prolonged CPB time is related to an increasing embolic load^[3,23]^. The pivotal role of surgery time in CABG patients has fully been understood in the literature^[23-25]^. Brown et al.^[3]^ reported that each 60-minute increase in CPB time associates with 30.5% increase in the embolic burden. This increase is more severe in patients with valve repair surgery (145.3% per hour). In this line, a multivariate analysis showed even 30-minute increments in CPB time acts as an independent risk factor for postoperative death significantly (OR=1.57)^[26]^. Bucerius et al.^[27]^ indicated that CPB time longer than 2 hours should also be considered as an independent risk factor for stroke (OR=1.42). Therefore, it is implied that the final outcomes after CABG surgery are significantly associated with CPB time. Mean CPB time varied between 74 and 101 minutes and the absolute mean CPB time difference between high MAP and low MAP groups is up to 10 minutes in the present study. However, there was no significant difference in mean CPB time between groups in all RCTs; low MAP groups had experienced greater mean CPB time in all studies^[16-18]^. So probably the great impact of prolonged CPB time on emboli formation interfered with the neuroprotective effect of low MAP. In addition, we should bear in mind that mean statistics can be highly affected by extreme values and are not appropriate statistics for highly skewed distributions. An analysis of 5,000 patients undergoing CABG showed that the distribution of CPB duration is positively skewed (0.18) and data is dispersed (coefficient of variation=40%), showing that 25% of surgeries lasted longer than 135 minutes (up to 643 minutes)^[26]^. In this study, each 30-minute increment in CPB time increased 1.53 times the risk of death, so it implies that final outcomes, such as death, stroke, POCD, are very sensitive to CPB time. In the RCTs included in our review, coefficient of variation (CV) of CPB time is mainly between 20 to 30%, implying that there is considerable dispersion in data. It is also clear that mean statistics is not a valid estimate of skewed data, while dispersed cases have a strong association with the final outcomes. Therefore, dispersity may be responsible for the fact that the neuroprotective effect of low MAP is not significant in the meta-analysis. It is suggested that insignificant difference between mean CPB time is not enough to compare the occurrence of POCD between groups, so patients also need to be stratified for the CPB time.

### The Role of Age

Increasing age is associated with a higher occurrence of POCD in the low MAP group, based on the meta-regression analysis. Patients may benefit more from high MAP protocol as they age. On the other hand, low MAP protocol did not offer any advantage over the high MAP group. The result must be interpreted carefully, because the mean age varied between 64.75 and 66.87 years among studies (mean difference: 2.12 years). This finding is supported by other studies showing that elderly may need high MAP during CPB^[6,7,19]^. However, a multivariable linear regression revealed that MAP was unrelated to POCD, and interactions between age and MAP area less than 50 mm Hg were significant, meaning that hypotension contributed significantly to POCD in the elderly^[28]^. 

### The Role of Follow-up Period

Cognitive status has been assessed in different time periods in the studies (2 days and 6 months). The follow-up period ranges from 2 days after cardiac surgery to 6 months in the current RCTs. Most studies have reported that the highest rates of POCD occurred during the first weeks following CABG surgery. It was also followed by a signiﬁcant decline in the incidence rate in the ﬁrst 6 months^[3,29,9,30]^. Cormack et al. demonstrated a pattern developed for reduction in POCD across all four measures in the first year after CPB surgery^[1]^. They also reported that there is a decline in half of the routinely used tests in the first week after CPB surgery, specifically in measures that assess psychomotor function^[1]^. Moreover, the practice effect indicates that the results of patients with POCD may be confounded by the results of patients who showed practice effects over multiple testing sessions^[2]^. However, the improvement in the declining trend in POCD after CABG surgery may be originated from the learning effect. Brown et al. reported that as time passes after CPB, a consistent reduction in the number of microemboli is seen^[3]^, which means that there may be another explanation for the declining trend in POCD. 

### The Possible Role of Confounders

Some part of discrepancy among studies may come from the variability in the definition of POCD^[31]^. However, Gold et al.^[16]^ and Charlson et al.^[17]^ used a cognitive decline determined *a priori* by a panel of experts in the postoperative score in comparison to the preoperative score that might be different from the commonly used criteria in other neurocognitive studies (1 SD decline). One SD decline in the postoperative score in comparison to the preoperative score is the most commonly used measure to define POCD in the literature. A fixed amount of decline for each patient is used exclusively in this criterion. Mahanna et al.^[31]^ found that this criterion estimates between the higher (20% drop) and lower impairment index rating estimates of cognitive decline. Therefore, one SD decline criterion may miss up to 30% of the occurrence of POCD in both groups^[31]^. Finally, the both low and high MAP groups have randomly been assigned, so this discrepancy may not be recognized as a source of bias between groups. Cognitive testing is still considered as the cornerstone for POCD measurement in the light of limitation of POCD definition. In addition, Gold et al.^[16]^ and Charlson et al.^[17]^ defined POCD if occurred in three or more tests; that is clearly distinct from that of most of the research conducted in neurocognitive assessment (in one or more tests)^[2]^. Therefore, the result must be interpreted cautiously because the neurocognitive criteria are strict.

Patients lost to follow-up ranged from 3.1% to 12.38% in the present study. Meta-regression analysis revealed a significant association between the occurrence of POCD among RCTs and loss to follow-up (MH log RR: 0.358, 95% CI: 0.030-0.687, *Z-*value 2.138, *P*-value=0.032). Loss to follow-up in Siepe et al.^[18]^ was 12.38% in very early assessment. The validity of studies in which more than 20% of patients are lost to follow-up is questionable^[32]^. After exclusion of the study of Siepe et al.^[18]^, there was also no significant association between low MAP and POCD. It is worth mentioning that MHRR decreased from 1.012 to 0.681 in favor of low MAP insignificantly. In current studies, the rate of loss to follow-up is not significantly different between groups in order to lessen the bias caused by a notable dropout rate.

There may be multiple factors contributing to POCD, but very few of them have been proven to have the remarkable effect on cognitive deficit as much as CPB time. CABG surgery with valve repair may put patients as a greater risk for the occurrence of POCD than isolated CABG surgery^[3]^. All RCTs excluded valve repair, so it is not a concern in the current meta-analysis. Comparison of on- and off-pump among patients in CABG groups has not shown significant differences in the occurrence of POCD. In this way, a meta-analysis revealed that there is no significant protective effect for off-pump CABG^[33,10]^. On-pump CABG surgery is used in all RCTs, so surgery technique may not confound the results. 

### Limitations of the Present Study

Several limitations may be considered for this meta-analysis. The studies have been conducted from 1995 to 2011, so did not succeed to contact authors to access the raw data. The skewness of CPB time would be calculated if we had access to median CPB time. The effect of low MAP on cognitive deficit after CPB surgery with focus on CPB time deserves further investigation and it is necessary to conduct homogeneous follow-up timing and consensual definition of cognitive deficit. 

However, Turner et al.^[34]^ argued that when at least two adequately powered studies are available in meta-analysis, underpowered studies often contribute little information, so the result is reliable. Large multicenter, randomized studies are needed to provide additional data.

## CONCLUSION

Meta-analysis showed that POCD must be considered as a common event among CABG patients. Older patients may benefit less from low MAP. The neuroprotective effect of low MAP on POCD was attenuated by the prolonged CPB time. Shorter CPB time is related to a lower occurrence of POCD. Low MAP may decrease the risk of POCD after CABG surgery if CPB time stratified rigorously between low MAP and high MAP patients. 

**Table:** 

Authors' roles & responsibilities	
FHK	Substantial contributions to the conception or design of the work; or the acquisition, analysis, or interpretation of data for the work; final approval of the version to be published
AS	Substantial contributions to the conception or design of the work; or the acquisition, analysis, or interpretation of data for the work; final approval of the version to be published
MRH	Substantial contributions to the conception or design of the work; or the acquisition, analysis, or interpretation of data for the work; final approval of the version to be published
